# MicroRNA-223 Regulates Retinal Function and Inflammation in the Healthy and Degenerating Retina

**DOI:** 10.3389/fcell.2020.00516

**Published:** 2020-06-26

**Authors:** Nilisha Fernando, Josephine H. C. Wong, Shannon Das, Catherine Dietrich, Riemke Aggio-Bruce, Adrian V. Cioanca, Yvette Wooff, Joshua A. Chu-Tan, Ulrike Schumann, Chinh Ngo, Rohan W. Essex, Camilla Dorian, Sarah A. Robertson, Si Ming Man, Jan Provis, Riccardo Natoli

**Affiliations:** ^1^The John Curtin School of Medical Research, The Australian National University, Canberra, ACT, Australia; ^2^ANU Medical School, The Australian National University, Canberra, ACT, Australia; ^3^Academic Unit of Ophthalmology, The Australian National University, Canberra, ACT, Australia; ^4^Robinson Research Institute, School of Medicine, The University of Adelaide, Adelaide, SA, Australia

**Keywords:** microRNA-223, retinal degeneration, macrophage, neuroinflammation, retinal function, photoreceptor cell death, microglia, microRNA

## Abstract

**Introduction:**

MicroRNAs (miRNAs) are small, non-coding RNA molecules that have powerful regulatory properties, with the ability to regulate multiple messenger RNAs (mRNAs) and biological pathways. MicroRNA-223-3p (miR-223) is known to be a critical regulator of the innate immune response, and its dysregulation is thought to play a role in inflammatory disease progression. Despite miR-223 upregulation in numerous neurodegenerative conditions, largely in cells of the myeloid lineage, the role of miR-223 in the retina is relatively unexplored. Here, we investigated miR-223 in the healthy retina and in response to retinal degeneration.

**Methods:**

miR-223-null mice were investigated in control and photo-oxidative damage-induced degeneration conditions. Encapsulated miR-223 mimics were intravitreally and intravenously injected into C57BL/6J wild-type mice. Retinal functional responses were measured using electroretinography (ERG), while extracted retinas were investigated by retinal histology (TUNEL and immunohistochemistry) and molecular analysis (qPCR and FACS).

**Results:**

Retinal function in miR-223^–/–^ mice was adversely affected, indicating that miR-223 may be critical in regulating the retinal response. In degeneration, miR-223 was elevated in the retina, circulating serum, and retinal extracellular vesicles. Conversely, retinal microglia and macrophages displayed a downregulation of miR-223. Further, isolated CD11b^+^ inflammatory cells from the retinas and circulation of miR-223-null mice showed an upregulation of pro-inflammatory genes that are critically linked to retinal inflammation and progressive photoreceptor loss. Finally, both local and systemic delivery of miR-223 mimics improved retinal function in mice undergoing retinal degeneration.

**Conclusion:**

miR-223 is required for maintaining normal retinal function, as well as regulating inflammation in microglia and macrophages. Further investigations are required to determine the targets of miR-223 and their key biological pathways and interactions that are relevant to retinal diseases. Future studies should investigate whether sustained delivery of miR-223 into the retina is sufficient to target these pathways and protect the retina from progressive degeneration.

## Introduction

Immune system dysregulation is a critical process involved in the onset and progression of retinal degenerative diseases (Ambati et al., 2013), including age-related macular degeneration (AMD), retinitis pigmentosa (RP), and diabetic retinopathy (DR). Subtle but chronic changes within the retinal microenvironment, such as the steady build-up of reactive oxygen species (ROS), can lead to a larger inflammatory response building in the retina (Xu et al., 2009). Microglia, the resident immune cells of the central nervous system (CNS), migrate to the site of injury or degeneration, where they may also recruit blood-borne macrophages (Penfold et al., 2001; Karlstetter et al., 2015), leading to cytokine secretion, complement system activation, and phagocytosis of degenerating and living photoreceptors (Zhao et al., 2015; Akhtar-Schafer et al., 2018; Silverman and Wong, 2018; Wooff et al., 2019). Understanding how abnormal immune responses can be controlled is therefore paramount in order to develop effective treatments for reducing inflammation in retinal diseases.

MicroRNAs (miRNAs) are highly conserved, small non-coding RNA molecules that are approximately 22 nucleotides in length that can regulate specific gene targets. By binding to the 3′ untranslated region (3′ UTR) of a messenger RNA (mRNA), the mRNA is targeted for translational repression or degradation (reviewed in Bartel, 2004). A single miRNA has the ability to regulate the expression of hundreds of mRNAs due to seed sequence similarity in the 3′ UTRs of many mRNAs (Bartel, 2004). Often, many targets of a single miRNA exist within associated or the same biological pathways; hence, miRNAs can be powerful regulators (Bartel, 2004). The immune system is extensively modulated by miRNA signaling, with many miRNAs well characterized to participate in inflammatory regulation (reviewed in O’Connell et al., 2012). Specifically within the retina, miRNAs are known to play a major role in the development and disease of the photoreceptors, bipolar cells, and Müller cells (Zuzic et al., 2019), including the regulation of retinal inflammation (Anasagasti et al., 2018; Chu-Tan et al., 2018).

In a systematic review of studies on miRNAs and neurodegenerative diseases (including Alzheimer’s disease, multiple sclerosis, Parkinson’s disease, amyotrophic lateral sclerosis, and AMD), miR-223-3p was identified as one of the top miRNAs upregulated in diseased conditions, alongside miR-146a-5p and miR-155-5p (Juźwik et al., 2019). Although both mature strands of miR-223 (miR-223-5p and miR-223-3p) are functional and each regulates a different set of mRNAs, miR-223-3p appears to be heavily associated with neurodegeneration (Juźwik et al., 2019). Therefore, from here on, miR-223-3p will be referred to as miR-223 and is the focus of this study. miR-223 is produced primarily within cells of the hematopoietic system, with the highest levels of expression observed in myeloid cells, particularly neutrophils and macrophages (reviewed in Haneklaus et al., 2013; Yuan et al., 2018). miR-223 is thought to be expressed by a “myeloid-like” gene under tight regulation during granulopoiesis (Fukao et al., 2007). In its mature form, miR-223 is known to regulate immune cell functions through several proposed mechanisms, including myeloid activation and regulation (Fazi et al., 2005; Gilicze et al., 2014; Galloway et al., 2019), NLRP3 inflammasome suppression (Bauernfeind et al., 2012; Yang et al., 2015; Neudecker et al., 2017b), IGF1R signaling (Qadir et al., 2015), cathepsin activity (Gantier, 2013), suppression of chemokines (Dorhoi et al., 2013), downregulation of the NF-κB pathway (Zhou et al., 2018), and alteration of JAK/STAT signaling (Chen et al., 2012). In the CNS, miR-223 may be a circulating biomarker in stroke (Wang et al., 2014) and multiple sclerosis (Fenoglio et al., 2013), and is also elevated in the plasma (Ertekin et al., 2014) and in peripheral blood cells (Litwinska et al., 2019) of neovascular AMD patients. In addition, exosomal miR-223 has been demonstrated to play an important role in cell signaling in stroke and dementia (Chen et al., 2017; Wei et al., 2018).

In the retina, elevated levels of miR-223 have also been found in models of experimental autoimmune uveoretinitis (EAU) (Watanabe et al., 2016), Müller cell disruption (Chung et al., 2016), amyloid-beta-induced retinal degeneration (Huang et al., 2017), optic nerve crush injury (Fuller-Carter et al., 2015), and in photo-oxidative damage (Saxena et al., 2015). However, the role that miR-223 plays in the healthy retina and in disease progression is unknown. Here, we conducted an exploratory investigation into the role of miR-223 in the retina under normal conditions and in a photo-oxidative damage model of retinal degeneration (Natoli et al., 2016a), using miR-223-null mice. We demonstrate that miR-223 has an important role in maintaining normal retinal function. We also show that miR-223 may modulate inflammation in retinal and circulating immune cells during disease.

## Materials and Methods

### Animal Handling

All experiments were conducted in accordance with the ARVO Statement for Use of Animals in Ophthalmic and Vision Research and with approval from the Australian National University (ANU) Animal Experimentation Ethics Committee (Ethics ID: A2017/41). Adult wild-type (WT) C57BL/6J mice (ANU Phenomics Facility) and miR-223-null mutant mice (miR-223^–/–^, B6.Cg-*Ptprc^a^ Mir223^tm1Fcam^*/J, #013198, Jax Laboratories) were utilized for all experiments. Animals were born and reared under normal lighting conditions (12:12-h light to dark cycle of ∼5 lux light) and were aged at 50–70 post-natal days at the time of use. All animals were screened for *Crb1*^rd8^, an inherited retinal degeneration present in some commercial lines, using primers described previously (Mattapallil et al., 2012) and were deemed free of this mutation (ANU Phenomics Facility and JCSMR Biomolecular Resource Facility). Sample sizes (N) are included in each figure legend.

### Separation of Retinal Microglia and Macrophages by FACS

We employed a fate-mapping strategy for the separation of retinal microglia and recruited monocyte-derived macrophages (CX3CR1^+^) by using fluorescence-activated cell sorting (FACS), according to a previously described model by O’Koren et al. (2016). In brief, tamoxifen (Sigma Aldrich, St. Louis, MO, United States) was dissolved in corn oil at 37°C at a concentration of 20 mg/ml. P60 CX3CR1^YFP–CreER/wt^:R26^RFP^ mice (ANU Phenomics Facility) were intraperitoneally (IP) injected with tamoxifen (75 mg/kg) twice, 24 h apart, to induce Cre recombinase and RFP expression in all CX3CR1^+^ cells. Following a “wash out” period of 60 days, mice were exposed to 5 days of photo-oxidative damage. In this model, the “wash out” period allows for RFP expression to be lost in circulating monocytes due to turnover, but RFP expression to be retained by long-lived resident microglia (O’Koren et al., 2016). Following photo-oxidative damage, CX3CR1^YFP–CreER/wt^:R26^RFP^ retinas were collected via corneal incision and digested using mechanical dissociation and papain digestion (LS003126; Worthington Biochemicals, Lakewood, NJ, United States), as described previously (Mulfaul et al., 2020; Wooff et al., 2020b). YFP^+^ microglia/macrophages were FACS-isolated based on relative expression of RFP (BD FACS Aria III; JCSMR Imaging and Cytometry Facility). Microglia are YFP^+^RFP^+^, whereas recruited macrophages are YFP^+^ only.

### Blood Collection and Preparation for FACS

Peripheral blood was collected to measure miR-223 in circulating serum and to isolate CD11b^+^ cells for examining their inflammatory status. A sub-mandibular blood collection method (Golde et al., 2005) was used to collect blood from the submandibular vein using a lancet (Goldenrod animal lancet 4 mm point length; MEDIpoint Inc, Mineola, NY, United States). To collect serum for miRNA extraction, 200 μl peripheral blood was collected into 1.5 ml Eppendorf tubes and left at room temperature for 30 min to clot. Samples were centrifuged at 1500 g for 10 min at 4°C, following which the separated serum was used for miRNA extraction.

For FACS isolation of CD11b^+^ cells, peripheral blood was collected into Microtainer MAP microtubes containing K2EDTA (#363706; BD, Franklin Lakes, NJ, United States); 100 μl of each blood sample was mixed with 900 μl of distilled water for exactly 20 s (to initiate lysis of erythrocytes) and then lysis was stopped with 100 μl of 10× phosphate-buffered saline (PBS). Samples were spun at 500 *g* for 5 min at 4°C, and the pellets were resuspended in 900 μl of distilled water for exactly 20 s, before adding 100 μl of 10× PBS and centrifuging at 500 *g* for 5 min at 4°C. Pellets were resuspended and stained using a PE anti-mouse/human CD11b antibody (1:500 in 1× PBS, clone M1/70, #101207; BioLegend, San Diego, CA, United States). After staining for 40 min, samples were sorted by FACS (BD FACS Aria III; JCSMR Imaging and Cytometry Facility) into 1× PBS, which was replaced by TRIzol (Thermo Fisher Scientific) for RNA extraction.

### Delivery of miR-223 Mimics

To achieve local and systemic transfection of synthetic miR-223 mimics, a miR-223-3p mimic (#MC12301, hsa-miR-223-3p; Thermo Fisher Scientific, Waltham, MA, United States) and a negative control mimic (#4464058; Thermo Fisher Scientific) were each encapsulated in Invivofectamine 3.0 (#IVF3001; Thermo Fisher Scientific) and sterile endotoxin-free 1× PBS (pH 7.4, Thermo Fisher Scientific) according to our previously published methods (Chu-Tan et al., 2020).

Wild-type animals were anesthetized using a mixture of Ketamine (100 mg/kg body weight; Troy Laboratories, Glendenning, NSW, Australia) and Ilium Xylazil-20 (12 mg/kg body weight; Troy Laboratories), delivered through IP injection. For retinal transfection, encapsulated mimics were intravitreally (IVT) injected at 1 μg/μl (1 μg per eye), according to our previously described methods (Chu-Tan et al., 2020). Injections were performed 3 h prior to photo-oxidative damage, with our previous study indicating that retinal transfection is effective for 3–4 days post-injection and can be detected in all layers of the retina (Chu-Tan et al., 2020).

For systemic intravenous (IV) delivery of mimics, tail vein injections were performed on restrained mice with the aid of a heat lamp. Each mouse received 0.5 mg/kg of encapsulated mimic, as recommended by the manufacturer. Injections were performed at 2 days into a 5-day photo-oxidative damage paradigm, as mimics may have a shorter half-life in circulation.

### Photo-Oxidative Damage

To induce retinal degeneration using photo-oxidative damage (PD), animals were subject to continuous white LED light exposure at 100 K lux for a period of either 1, 3, 5, or 7 days, according to our previously described pigmented mouse model (Natoli et al., 2016a). Animals were administered pupil dilator eye drops twice daily (1% w/v Minims atropine sulfate; Bausch and Lomb, Garden City, NY, United States). Dim-reared control animals with no photo-oxidative damage (12:12-h light to dark cycle of ∼5 lux light) were used for comparison.

### Electroretinography

Electroretinography (ERG) was used to measure retinal function in mice in response to full-field flash stimuli under scotopic conditions (with dark adaptation for approximately 16 h). A single-flash paradigm was used to elicit mixed (rod and cone) responses, over an intensity range of -2.0 to 1.6 log cd.s/m^2^ using the Celeris full-field ERG system (Diagnosys LLC, Lowell, MA, United States). Measurements of the amplitudes of the a-wave (photoreceptor activity) and b-wave (ON-bipolar and Müller cell activity) were performed as an assessment of retinal function using Espion V6 software (Diagnosys LLC).

### Histological Analysis of Retinal Cryosections

Following euthanasia of the animal using carbon dioxide, whole eyes were enucleated and cryosectioned at 12 μm in the parasagittal plane for histological analysis (CM1850; Leica, Wetzlar, Germany). To detect photoreceptor cell death in the outer nuclear layer (ONL), the terminal deoxynucleotidyl transferase (Tdt) dUTP nick end labeling (TUNEL) assay (Roche Diagnostics, Basel, Switzerland) was used on retinal cryosections, according to previously described protocols (Maslim et al., 1997; Natoli et al., 2010). Sections were counterstained using a DNA label (Bisbenzimide or Hoechst’s stain, DAPI, 1:10,000; Sigma Aldrich) for visualization of the cellular layers and the retinal pigment epithelium (RPE).

For immunohistochemistry in retinal cryosections, antigen retrieval (ImmunoSolutions, Brisbane, QLD, Australia) was performed prior to incubation with primary antibodies. To visualize the migration and recruitment of microglia and macrophages to the outer retina, a primary antibody against ionized calcium-binding adaptor molecule 1 (rabbit α-IBA1, 1:500, #019-19741; Wako, Osaka, Japan) was used according to previously described protocols (Rutar et al., 2011b, 2015). A primary antibody against glial fibrillary acidic protein (rabbit α-GFAP, 1:500, #Z0334; Dako, Agilent, Santa Clara, CA, United States) was used to determine reactive gliosis, with staining localized to Müller cells and astrocytes. Sections were counterstained with a DNA label (DAPI) for visualization. Negative controls were incubated with no primary antibody.

TUNEL^+^ photoreceptors and IBA1^+^ cell counts in the outer retina (ONL-RPE) were performed along the full length of retinal cryosections (supero-inferior) in duplicate. The number of rows of photoreceptor cell nuclei was quantified in the superior retina (approximately 1 mm superior to the optic nerve) to determine ONL thinning, as described previously (Fernando et al., 2016). For each section, three measurements were taken, and sections were counted in duplicate.

For GFAP staining, immunofluorescence was quantified in each cryosection using two methods. Firstly, the ratio of the GFAP staining area to the whole retinal area was taken. Each image was binarized in ImageJ (NIH, Bethesda, MD, United States). The threshold function was then used to isolate the stained area. The area of GFAP staining, as well as the whole retinal area between the inner limiting membrane (ILM) and the outer limiting membrane (OLM), as observed through DAPI staining of the cellular layers, was graphed as a ratio. This represents GFAP staining in Müller cells and astrocytes in the whole retina. Secondly, as GFAP staining is localized to both Müller cells and astrocytes located in the inner retina, GFAP levels can be very intense between the ILM and the ganglion cell layer (GCL). Fluorescence intensity in the other layers of the retina was determined as an indication of GFAP staining in the Müller cell processes. This was performed by determining the intensity of staining in all layers between the inner plexiform layer (IPL) and the OLM in ImageJ. This represents GFAP staining in the Müller cell processes through all layers between the IPL and the OLM in the retina.

### Primary Cell Cultures

Primary retinal CD11b^+^ microglia were isolated from dim-reared mouse retinas and sorted by FACS (BD FACS Aria III; JCSMR Imaging and Cytometry Facility) into a 48-well plate at ∼1500 cells per well, as previously described (Mulfaul et al., 2020; Wooff et al., 2020b). Cells were stained with a PE anti-mouse/human CD11b antibody (clone M1/70, #101207; BioLegend) for 40 min prior to FACS. Isolated primary microglia were cultured for 4 weeks in Dulbecco’s Modified Eagle Medium (DMEM)-F12 (Thermo Fisher Scientific) supplemented with 10% fetal bovine serum (FBS; Sigma Aldrich), 1% antibiotic-antimycotic (Thermo Fisher Scientific), 3% L-glutamine (Thermo Fisher Scientific), 0.25 ng/ml GM-CSF (Stem Cell Technologies, Vancouver, BC, Canada), and 2.5 ng/ml M-CSF (Miltenyi Biotec, Bergisch Gladbach, Germany), as previously described (Mulfaul et al., 2020; Wooff et al., 2020b).

Primary bone marrow-derived macrophages (BMDMs) were extracted from the hind leg bone marrow of wild-type dim-reared mice and were cultured for 6 days in DMEM (high glucose with sodium pyruvate; Thermo Fisher Scientific) supplemented with 10% FBS (Sigma Aldrich), 1% L-glutamine (Thermo Fisher Scientific), 30% L929-conditioned media, 1% penicillin and streptomycin (Thermo Fisher Scientific), and 1% non-essential amino acids (Thermo Fisher Scientific), as described previously (Man et al., 2016; Fox et al., 2020). Cells were seeded at 0.5 × 10^6^ cells/well in a 24-well plate in antibiotic-free media 24 h prior to experimentation.

### Immortalized Cell Cultures

Immortalized cell lines for photoreceptor-like cells [mouse 661W; Dr. Muayyad R. Al-Ubaidi, University of Oklahoma Health Sciences Centre, Oklahoma City, OK, United States (al-Ubaidi et al., 1992)], RPE-like cells [human ARPE-19, CRL-2302; ATCC, Manassas, VA, United States (Dunn et al., 1996)], microglia-like cells [mouse C8B4, CRL-2540; ATCC (Alliot et al., 1996)], and Müller-like cells [human MIO-M1; Moorfield’s Institute of Ophthalmology, London, United Kingdom (Limb et al., 2002)] were also used in this study. Immortalized cell lines were authenticated by CellBank (Sydney, Australia) and were cultured with DMEM (high glucose with sodium pyruvate; Thermo Fisher Scientific) supplemented with 10% FBS (Sigma Aldrich), 1% antibiotic-antimycotic (Thermo Fisher Scientific), and 3% L-glutamine (Thermo Fisher Scientific). Cells were incubated at 37°C in a humidified atmosphere (5% CO_2_).

Cells were seeded in 24-well plates at varying densities (661W at 2.5 × 10^4^ cells/well, ARPE19 at 10 × 10^4^ cells/well, C8B4 at 15 × 10^4^ cells/well and MIO-M1 at 2 × 10^4^ cells/well) and were incubated in reduced serum (1% FBS) DMEM media with additives, as described above, for 24 h prior to the start of experiments.

### *In vitro* Stimulation for Oxidative Stress and Inflammation

661W photoreceptor-like cells were stimulated with 15,000 lux white light exposure for 5 h, according to previously described protocols (Natoli et al., 2016b). Cells with no light exposure were used as unstimulated controls. C8B4 cells, primary retinal CD11b^+^ microglia, and primary BMDMs were stimulated with 20 ng/ml lipopolysaccharide (LPS from *E. coli* 0111:B4, #N4391; Sigma Aldrich) for 4 h, followed by 5 mM adenosine triphosphate (ATP, #A6419; Sigma Aldrich) for 0.5 h. MIO-M1 cells were stimulated with 10 ng/ml TNF-α (#210-TA; R&D Systems, Minneapolis, MN, United States) for 24 h, followed by 5 mM ATP for 0.5 h. ARPE19 cells were stimulated with 50 ng/ml recombinant human interleukin-1α (IL-1α) protein (#ab9615; Abcam, Cambridge, United Kingdom) for 24 h, followed by 5 mM ATP for 0.5 h. Unstimulated cells were used as controls for comparison within each cell type. For sample collection, cells were washed in 1× PBS, then lysed and homogenized in TRIzol reagent (Thermo Fisher Scientific), prior to RNA extraction.

### Isolation of Retinal Extracellular Vesicles

For molecular analysis, retinas were excised using corneal incision. For RNA extraction from retinal extracellular vesicles, each sample contained two pooled retinas (from one mouse). Isolation of small-medium extracellular vesicles (s-mEVs) from papain-digested retinas was performed according to previously published methodology (Wooff et al., 2020a) and in accordance with the MISEV2018 guidelines (Théry et al., 2018). Characterization of the properties of isolated retinal s-mEVs (which include exosomes) has been published, detailing transmission electron microscopy, size distribution analysis, and western blot analysis of these s-mEVs (Wooff et al., 2020a).

### qPCR for miR-223 and Inflammatory Genes

Isolated retinas, serum, retinal s-mEVs, and cell lysates were processed for RNA extraction using a combination of TRIzol (Thermo Fisher Scientific) and a RNAqueous Micro Total RNA Isolation Kit (Thermo Fisher Scientific), or a *mir*Vana miRNA Isolation Kit (Thermo Fisher Scientific), according to the manufacturer’s protocol and our previously described protocols (Natoli et al., 2008). RNA samples were tested for purity and concentration using an ND-1000 spectrophotometer (Nanodrop Technologies, Wilmington, DE, United States; JCSMR Biomolecular Resource Facility). Samples were stored at -80°C.

cDNA was prepared from 250 to 500 ng RNA (dependent on sample type) using a Tetro cDNA Synthesis Kit (Bioline, London, United Kingdom), according to the manufacturer’s protocol. For miRNA conversion to cDNA, a TaqMan miRNA Reverse Transcription Kit (Thermo Fisher Scientific) was used with specific RT primers from TaqMan miRNA assays (refer to Table 1).

**TABLE 1:** TaqMan assays used for qPCR (Thermo Fisher Scientific).

**Gene symbol**	**Gene name**	**Catalog number**
*C1qa*	Complement component 1, q subcomponent, alpha polypeptide	Mm00432142_m1
*C3*	Complement component 3	Mm00437858_m1
*Ccl3*	Chemokine (C-C motif) ligand 3	Mm00441259_g1
*Ctse*	Cathepsin E	Mm00456010_m1
*Gapdh*	Glyceraldehyde-3-phosphate dehydrogenase	Mm99999915_g1
*Il-1*β	Interleukin-1β	Mm00434228_m1
*Nlrp3*	NLR family pyrin domain containing 3	Mm00840904_m1
*Stat3*	Signal transducer and activator of transcription 3	Mm01219775_m1
miR-223	miR-223-3p	#002295
U6	U6 snRNA	#001973

Quantitative real-time polymerase chain reaction (qPCR) was performed to measure gene and miRNA expression using a combination of cDNA, TaqMan Gene Expression Master Mix (Thermo Fisher Scientific) and TaqMan assays (refer to Table 1) according to the manufacturer’s protocol. qPCR reactions were performed in duplicate in a 384-well plate format and run using a QuantStudio 12K Flex instrument and software (Thermo Fisher Scientific; JCSMR Biomolecular Resource Facility). Analysis was performed using the comparative cycle threshold (CT) method (ΔΔCT), which was normalized to the *Gapdh* reference gene. miR-223 expression was normalized to U6 expression.

Genes for pro-inflammatory panels were selected on the following basis: (1) *Ccl3*, *Nlrp3*, and *Stat3* are validated targets of miR-223-3p (Bauernfeind et al., 2012; Chen et al., 2012; Dorhoi et al., 2013) and are known to be involved in retinal degenerative diseases (Doyle et al., 2012; Kohno et al., 2014; Chen et al., 2016, 2019; Fernando et al., 2016; Wooff et al., 2020b); (2) we have shown that *C1qa*, *C3*, and *Il-1*β are all critically linked to retinal disease progression in photo-oxidative damage (Natoli et al., 2017a, b; Jiao et al., 2018); and (3) *Ctse* is a predicted target of miR-223-3p and is involved in key inflammatory cell functions (Gantier, 2013).

### Imaging and Statistical Analysis

Fluorescence labeling of retinal cryosections was visualized and images were acquired using either an LSM800 confocal microscope and software (ZEISS, Oberkochen, Germany) or a Nikon A1^+^ confocal microscope and software (Nikon, Tokyo, Japan). Images were processed using ImageJ. All graphing and statistical analyses were performed using Prism 6 (GraphPad Software, La Jolla, CA, United States). Significance testing was performed using unpaired Student’s *t*-tests (*P* < 0.05). Significance testing of ERG datasets was analyzed using two-way analysis of variance (ANOVA) with *post hoc* Sidak’s multiple comparisons test to determine statistical significance (*P* < 0.05). Graphs were generated showing mean ± SEM values.

## Results

### miR-223 Signaling in the Normal Retina

**FIGURE 1:**
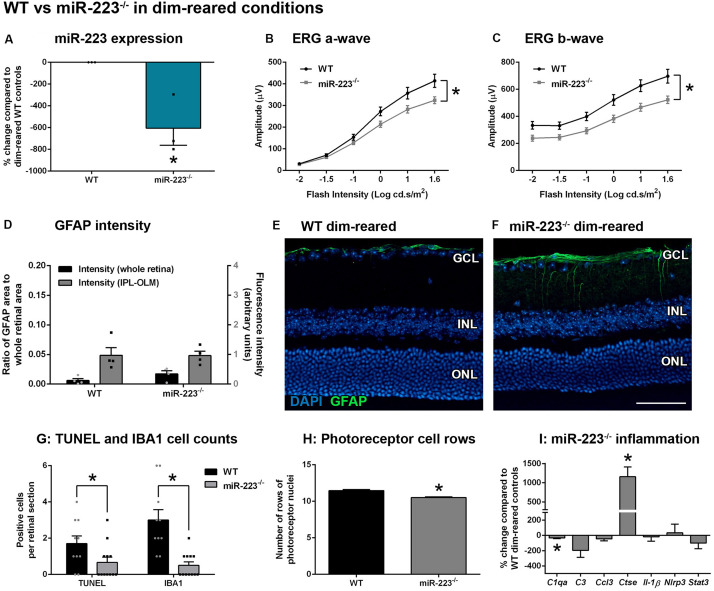
Retinas of wild-type (WT) and miR-223^–/–^ mice under normal, dim-reared conditions. **(A)** miR-223^–/–^ mice were confirmed to have a significant reduction in retinal miR-223 expression compared to WT mice (*P* < 0.05). **(B,C)** Electroretinography (ERG) of the a-wave **(B)** and b-wave **(C)** demonstrated that miR-223^–/–^ mice had reduced retinal function compared to WT controls (*P* < 0.05). **(D–F)** No significant differences in GFAP levels (green) were observed between groups (*P* > 0.05). Units for whole retinal GFAP on left *Y*-axis (black) and units for IPL to OLM GFAP on right *Y*-axis (gray). **(G)** TUNEL^+^ and IBA1^+^ cells were reduced in the outer retina of miR-223^–/–^ mice compared to WT controls (*P* < 0.05). **(H)** However, miR-223^–/–^ retinas had more photoreceptor layer thinning than WT retinas (*P* < 0.05). **(I)** *C1qa* and *Ctse* retinal expressions were significantly changed in miR-223^–/–^ retinas compared to WT controls (*P* < 0.05). Expression of *C3*, *Ccl3*, *Il-1*β, *Nlrp3*, and *Stat3* were unchanged (*P* > 0.05). IPL, inner plexiform layer; OLM, outer limiting membrane; GCL, ganglion cell layer; INL, inner nuclear layer; ONL, outer nuclear layer. Scale bar is 50 μm. Sample size is *N* = 3–6. *Denotes significance (*P* < 0.05).

We sought to determine whether miR-223 plays a regulatory role in the retina under normal conditions. We used dim-reared adult miR-223-null (miR-223^–/–^) mice that were confirmed to have decreased miR-223 expression, compared to wild-type (WT) age-matched dim-reared control mice (Figure 1A, *P* < 0.05). When investigating the retinal response, miR-223^–/–^ animals demonstrated a significantly reduced ERG a-wave (Figure 1B, *P* < 0.05), indicating less photoreceptor function, and a significantly decreased ERG b-wave (Figure 1C, *P* < 0.05), indicative of reduced ON-bipolar and Müller cell activity. This indicates that an absence of miR-223 may contribute to disruptions in normal retinal function. We investigated reactive gliosis by staining for the marker GFAP, which showed no significant change in the intensity or area of GFAP staining between miR-223^–/–^ and WT groups (Figures 1D–F, *P* > 0.05). Although miR-223^–/–^ retinas showed significantly reduced TUNEL^+^ photoreceptors and IBA1^+^ cells in the outer retina compared to WT controls (Figure 1G, *P* < 0.05), overall miR-223^–/–^ retinas showed more cumulative photoreceptor layer thinning (Figure 1H, *P* < 0.05). qPCR for a suite of pro-inflammatory cytokines, complement components, and innate immune system contributors [several of which are targets of miR-223 regulation (Bauernfeind et al., 2012; Chen et al., 2012; Dorhoi et al., 2013; Gantier, 2013)] was performed on whole retinal tissue. It was demonstrated that there was a small but significant decrease in complement component 1qa (*C1qa*) and significantly increased expression of cathepsin E (*Ctse*) in miR-223^–/–^ retinas (Figure 1I, *P* < 0.05).

### miR-223 Production in Retinal Damage

**FIGURE 2:**
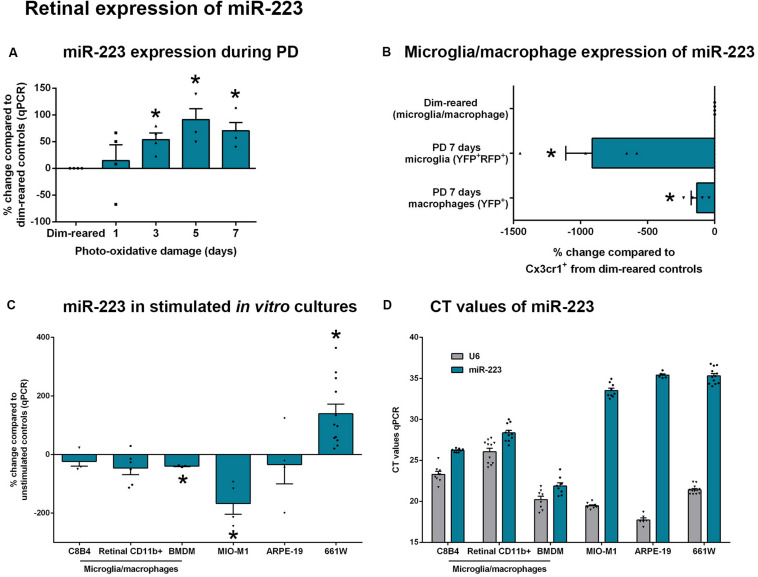
Retinal expression of miR-223 during stimulation. **(A)** During photo-oxidative damage (PD) in wild-type (WT) mice, miR-223 in the retina is significantly upregulated between 3 and 7 days, peaking at 5 days (*P* < 0.05), compared to dim-reared control levels. **(B)** Compared to dim-reared microglia/macrophages (Cx3cr1^+^ cells), isolated microglia (YFP^+^RFP^+^) and macrophages (YFP^+^) from 7-day PD mice had significantly reduced miR-223 levels (*P* < 0.05). **(C)** BMDMs and MIO-M1 cells had significantly reduced miR-223 after stimulation in culture, whereas 661W cells showed an increase in miR-223 (*P* < 0.05). C8B4 cells, retinal CD11b^+^ cells, and ARPE-19 cells showed no change in the expression of miR-223 between conditions (*P* > 0.05). **(D)** When comparing the CT values of miR-223 and U6 (reference) across cell types in both unstimulated and stimulated conditions, it is shown that microglia/macrophages have a higher expression of miR-223 (lower CT) relative to other cell types (higher CT of 34–36 indicates low expression of miR-223). Sample size is *N* = 3–12. *Denotes significance (*P* < 0.05).

miR-223 is thought to be primarily produced by cells of the myeloid lineage in many tissues, particularly in neutrophils and macrophages (Haneklaus et al., 2013). However, its expression has not yet been characterized in retinal degenerative conditions. We investigated the expression of miR-223 using a photo-oxidative damage model of retinal degeneration, in which central, focal photoreceptor degeneration and loss of retinal function are elicited (Natoli et al., 2016a). Using this model, we have demonstrated that the presence of outer retinal microglia and macrophages within the photoreceptor layer and subretinal space is critically linked with progressive degeneration and lesion expansion (Natoli et al., 2017a). Here, we showed that following 3–7 days of photo-oxidative damage, levels of miR-223 are substantially increased in WT retinas compared to dim-reared WT control retinas (Figure 2A, *P* < 0.05). There was a ∼100% increase at 5 days light exposure, which is concurrent with a peak in cell death and outer retinal IBA1^+^ microglia and macrophages in this model (Natoli et al., 2016a).

We then sought to determine the modulation in expression levels of miR-223 within retinal cell types both *in vivo* and *in vitro*. As miR-223 is thought to be primarily expressed by cells of the myeloid lineage (Haneklaus et al., 2013), we used a fate-mapping strategy that distinguishes resident microglia from recruited macrophages (O’Koren et al., 2016), as these are the main classes of immune cells present in the degenerating retina. Microglia and macrophages from photo-oxidative damaged retinas were compared to dim-reared control microglia/macrophages. This reference control population contains primarily microglia, as the retina contains few macrophages without damage (Natoli et al., 2017a). On a per-cell basis, FACS-isolated microglia (YFP^+^RFP^+^) and macrophages (YFP^+^) both have reduced miR-223 levels at 7 days photo-oxidative damage, compared to dim-reared control cells (Figure 2B, *P* < 0.05). Further, resident microglia isolated from 7-day photo-oxidative damaged retinas showed a larger decrease in miR-223 than recruited macrophages at the same time point (Figure 2B).

*In vitro*, stimulated cultures of microglia and macrophages (C8B4 microglia, retinal CD11b^+^ cells and BMDMs) all showed reduced miR-223 compared to unstimulated controls (Figure 2C), with BMDMs showing a significant change (*P* < 0.05). Müller cell cultures (MIO-M1) also showed a significant decrease in miR-223 under stimulated conditions (Figure 2C, *P* < 0.05), whereas ARPE-19 cells showed no change in miR-223 expression. Interestingly, a significant increase in miR-223 was detected in 661W photoreceptor-like cells (Figure 2C, *P* < 0.05) after stimulation with light. However, when investigating the CT values of miR-223 expression between cell types in both unstimulated and stimulated conditions, only microglia and macrophages showed robust expression of miR-223 (lower CT values for miR-223, Figure 2D). MIO-M1, ARPE-19, and 661W cells demonstrated only low miR-223 expression (high CT values of 34–36, Figure 2D). Therefore, in this *in vitro* comparison, microglia and macrophages were heavily abundant in miR-223 compared to Müller, RPE, and photoreceptor cell types.

Taken together, these data indicate that retinal miR-223 increases in photo-oxidative damage. Although microglia and macrophages are likely to be the primary producers of miR-223 in the retina, their expression of miR-223 decreases on a per-cell basis in retinal degeneration. Photoreceptors may also contribute towards an increase in miR-223 levels seen in retinal degeneration.

### The Role of miR-223 in Retinal Degeneration

**FIGURE 3:**
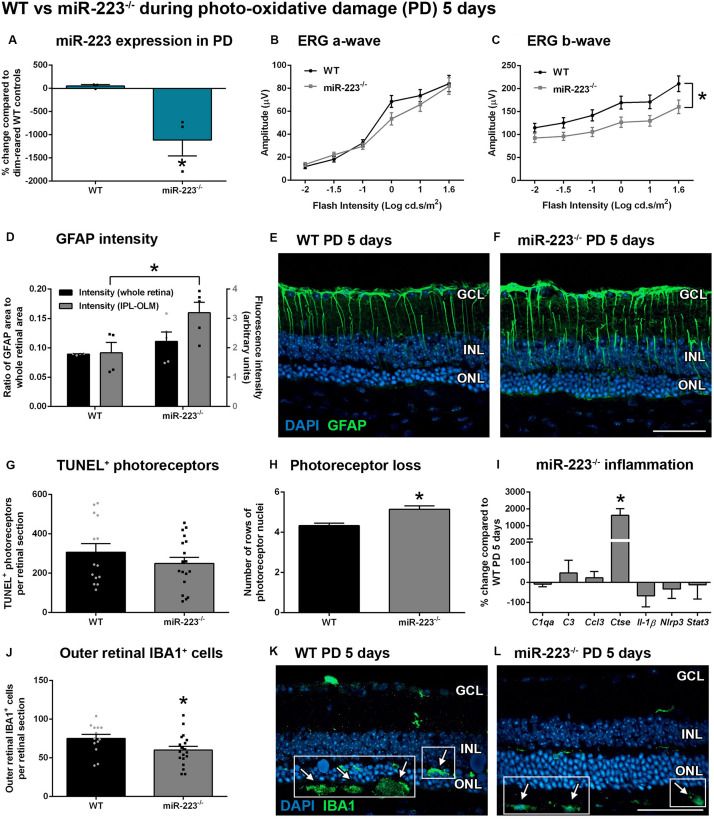
Retinas of wild-type (WT) and miR-223^–/–^ mice during 5 days of photo-oxidative damage (PD). **(A)** miR-223^–/–^ mice were again confirmed to have a significant reduction in retinal miR-223 expression compared to WT mice (*P* < 0.05). **(B,C)** Electroretinography (ERG) demonstrated that there was no change in the a-wave [**(B)**, *P* > 0.05], but a significant change in the b-wave [**(C)**, *P* < 0.05] in miR-223^–/–^ mice compared to WT controls. **(D–F)** GFAP intensity (green) was higher in all layers between the IPL and the OLM of miR-223^–/–^ retinas compared to WT retinas (*P* < 0.05). Units for whole retinal GFAP on left *Y*-axis (black) and units for IPL to OLM GFAP on right *Y*-axis (gray). **(G)** No change in TUNEL was observed between groups (*P* > 0.05). **(H)** miR-223^–/–^ retinas had more photoreceptor cell rows than WT retinas (*P* < 0.05), indicating less cumulative photoreceptor death. **(I)** *Ctse* retinal expression was significantly increased in miR-223^–/–^ retinas compared to WT controls (*P* < 0.05); however, no changes in any other inflammatory genes assayed were observed (*P* > 0.05). **(J–L)** miR-223^–/–^ retinas had a significant reduction in IBA1^+^ (green) microglia and macrophages in the outer retina (arrows, white boxes), compared to WT controls (*P* < 0.05). These were mainly present in the subretinal space. In WT animals, IBA1^+^ cells were increased in number and situated closer to the photoreceptor layer (arrows, white boxes). IPL, inner plexiform layer; OLM, outer limiting membrane; GCL, ganglion cell layer; INL, inner nuclear layer; ONL, outer nuclear layer. Scale bar is 50 μm. Sample size is *N* = 3–9. *Denotes significance (*P* < 0.05).

As miR-223 levels were shown to peak at 5 days during photo-oxidative damage (Figure 2A), we next looked at the role of miR-223 in retinal degeneration at this time point using miR-223^–/–^ mice and age-matched WT control mice. Again, we confirmed a decrease in miR-223 expression in miR-223^–/–^ animals compared to WT controls (Figure 3A, *P* < 0.05). However, after 5 days of photo-oxidative damage, there was no difference in the ERG a-wave between groups (Figure 3B, *P* > 0.05), unlike dim-reared controls. Earlier, it was shown that dim-reared miR-223^–/–^ animals already had a lower a-wave than WT controls (Figure 1B). The data shown in Figure 3B indicate that after damage, WT animals had a faster decline in the a-wave response compared to miR-223^–/–^ animals. However, there was still a significant decrease in the ERG b-wave in miR-223^–/–^ animals compared to WT controls after photo-oxidative damage (Figure 3C, *P* < 0.05), consistent with the change in the b-wave in dim conditions (Figure 1C).

An assessment of reactive gliosis using the GFAP marker demonstrated that there was a significant increase in GFAP intensity in the Müller cell processes (all layers between the IPL and OLM) in miR-223^–/–^ retinas compared to WT controls (Figures 3D–F, *P* < 0.05). However, the area of GFAP staining was unchanged between WT and miR-223^–/–^ in whole retinas (Figure 3D, *P* > 0.05). Although there was no difference in TUNEL^+^ photoreceptors undergoing cell death (Figure 3G, *P* > 0.05), cumulatively there were more rows of photoreceptor cells present in the miR-223^–/–^ retinas compared to WT controls, indicating lower levels of photoreceptor loss in the knockout animals (Figure 3H, *P* < 0.05). Upon investigating the inflammatory status of the retina, it was found that there were significantly less IBA1^+^ microglia and macrophages in the outer retinas of miR-223^–/–^ mice compared to WT controls (Figures 3J–L, *P* < 0.05). In WT animals, there were more IBA1^+^ cells present closer to the photoreceptor layer (Figure 3K), whereas in miR-223^–/–^ animals, these were situated closer to the RPE and were fewer in number (Figure 3L). qPCR for a range of pro-inflammatory cytokines, complement components, and innate immune system contributors [several of which are targets of miR-223 regulation (Bauernfeind et al., 2012; Chen et al., 2012; Dorhoi et al., 2013; Gantier, 2013)] only revealed a significant increase in *Ctse* in miR-223^–/–^ retinas (Figure 3I, *P* < 0.05).

### miR-223 in the Retina and Circulation in Retinal Degeneration

**FIGURE 4:**
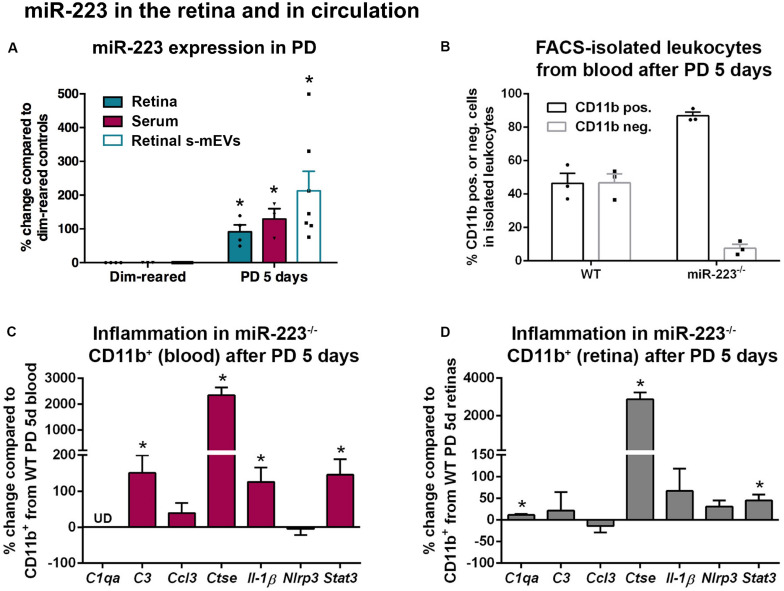
miR-223 in the retina and circulation during retinal degeneration. **(A)** miR-223 was shown to be significantly increased in the retina, serum, and retinal small-medium extracellular vesicles (s-mEVs) at 5 days of photo-oxidative damage (PD), compared to dim-reared control retinas, serum, and s-mEVs, respectively (*P* < 0.05). **(B)** During FACS-isolation of leukocytes after 5 days of PD, the number of CD11b^+^ cells to CD11b^–^ cells was skewed in the miR-223^–/–^ mouse blood compared to wild-type (WT) mouse blood, where the proportion between positive and negative populations was more even. **(C)** In CD11b^+^ cells isolated from blood after PD, *C3*, *Ctse*, *Il-1*β, and *Stat3* were all significantly upregulated compared to CD11b^+^ cells from WT (*P* < 0.05). The expression of *C1qa* was undefined (UD). **(D)** In the retina, CD11b^+^ cells had a significantly higher expression of *C1qa*, *Ctse*, and *Stat3* after PD compared to WT retinal CD11b^+^ cells (*P* < 0.05). Sample size is *N* = 3–7. *Denotes significance (*P* < 0.05).

Circulating and s-mEV levels of miR-223 have been shown to be dysregulated in several neurological disorders (Fenoglio et al., 2013; Wang et al., 2014; Chen et al., 2017; Wei et al., 2018). We assessed whether miR-223 was changed in response to photo-oxidative damage in both serum and retinal s-mEVs. We found that alongside elevated levels of retinal miR-223 at 5 days of photo-oxidative damage, there was also a significant increase in serum miR-223, compared to dim-reared controls (Figure 4A, *P* < 0.05). Retinal s-mEVs isolated at 5 days of photo-oxidative damage also showed an elevation in miR-223, compared to s-mEVs from dim-reared controls (Figure 4A, *P* < 0.05).

Circulating CD11b^+^ and CD11b^–^ leukocytes were then isolated from peripheral blood by FACS following 5 days of photo-oxidative damage (Figure 4B). While approximately equal amounts of CD11b^+^ and CD11b^–^ cells were identified in the blood of WT animals (mean of 46.4 and 46.8%, respectively, of cells within the leukocyte gate), miR-223^–/–^ animals had a large increase in the ratio of CD11b^+^ cells to CD11b^–^ cells (mean of 86.8 and 7.5%, respectively). This may indicate that miR-223^–/–^ animals undergoing photo-oxidative damage have a higher proportion of circulating CD11b^+^ cells compared to WT controls, potentially due to either increased CD11b^+^ cell generation, or loss of CD11b^–^ cells as a consequence of miR-223 deficiency.

Next, we quantified levels of pro-inflammatory cytokines, complement components, and innate immune system contributors by qPCR in CD11b^+^ cells isolated from blood (Figure 4C). In circulating CD11b^+^ cells from miR-223^–/–^ mice, there was a significant increase in complement component 3 (*C3*), *Ctse*, interleukin-1β (*Il-1*β), and signal transducer and activator of transcription 3 (*Stat3*) compared to WT controls (Figure 4C, *P* < 0.05). Pro-inflammatory genes *C3* and *Il-1*β are associated with progressive retinal degeneration in this model (Natoli et al., 2017a, b). Chemokine C-C motif ligand 3 (*Ccl3*) and NLR family pyrin domain containing 3 (*Nlrp3*) did not change (Figure 4C, *P* > 0.05), and *C1qa* was undetected in circulating CD11b^+^ cells.

From photo-oxidative damaged retinas, CD11b^+^ cells (primarily retinal microglia and macrophages) were also isolated for qPCR. *C1qa*, *Ctse*, and *Stat3* were significantly upregulated in CD11b^+^ cells isolated from miR-223^–/–^ retinas compared to WT retinas (Figure 4D, *P* < 0.05). No significant changes in *C3*, *Ccl3*, *Il-1*β, or *Nlrp3* were observed (Figure 4D, *P* > 0.05).

These data demonstrate that levels of miR-223 increase in the retina, circulation, and retinal s-mEVs in response to photo-oxidative damage. When no systemic miR-223 is present, the circulating inflammatory cell profile may be significantly altered towards a more pro-inflammatory activation state.

### Delivery of miR-223 Mimics to the Degenerating Retina

**FIGURE 5:**
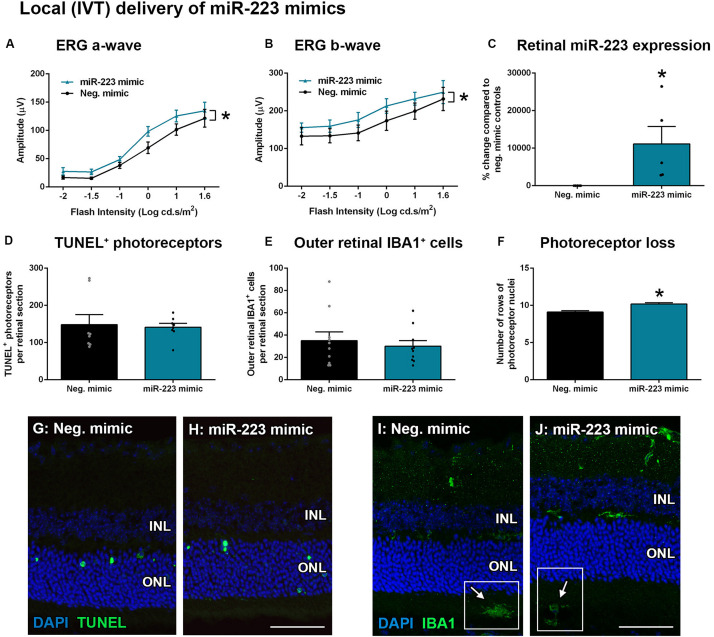
Local intravitreal (IVT) delivery of miR-223 mimics to the retina during 5 days of photo-oxidative damage. **(A,B)** WT animals injected with miR-223 mimics had a significantly higher a-wave **(A)** and b-wave **(B)** than negative controls (*P* < 0.05), as assessed by electroretinography (ERG). **(C)** Retinal expression of miR-223 was significantly higher at 5 days after mimics were intravitreally injected (*P* < 0.05). **(D,E)** There was no significant difference in TUNEL^+^ **(D)** or IBA1^+^ cells **(E)** in the outer retina between groups (*P* > 0.05). **(F)** Retinas injected with miR-223 mimics had a significantly higher number of photoreceptor rows compared to controls (*P* < 0.05). **(G,H)** Representative images depicting TUNEL^+^ photoreceptors (green). **(I,J)** Representative images depicting IBA1 immunohistochemistry (green), with arrows pointing towards IBA1^+^ cells in the subretinal space (white boxes). INL, inner nuclear layer; ONL, outer nuclear layer. Scale bar is 50 μm. Sample size is *N* = 4–5. *Denotes significance (*P* < 0.05).

Transfection of encapsulated miR-223 mimics into the retina via intravitreal (IVT) delivery, as well as systemic via tail vein (IV) delivery, was used to increase the bioavailability of miR-223 in the circulating or retinal system. As retinal microglia and recruited macrophages both contribute toward progressive photoreceptor loss in degeneration (Natoli et al., 2017a), we investigated the injection of miR-223 both locally and systemically to supplement these cells, as they lose miR-223 expression during degeneration (Figure 2). WT animals were IVT-injected with 1 μg/μl into each eye immediately prior to photo-oxidative damage. Following 5 days photo-oxidative damage, the ERG a-wave and b-wave were both significantly increased after injection with the miR-223 mimic, compared to scrambled mimic negative controls (Figures 5A,B, *P* < 0.05), indicating a positive effect on the retinal response. The retinal level of miR-223 was significantly increased at 5 days of photo-oxidative damage at approximately 10,000% higher than negative controls (Figure 5C, *P* < 0.05). No difference in TUNEL^+^ photoreceptors or outer retinal IBA1^+^ microglia and macrophages was observed (Figures 5D,E,G J; *P* > 0.05). However, there was a small but significant increase in the number of photoreceptor cell rows following injection with miR-223 mimics (Figure 5F, *P* < 0.05), consistent with an improved retinal response.

**FIGURE 6:**
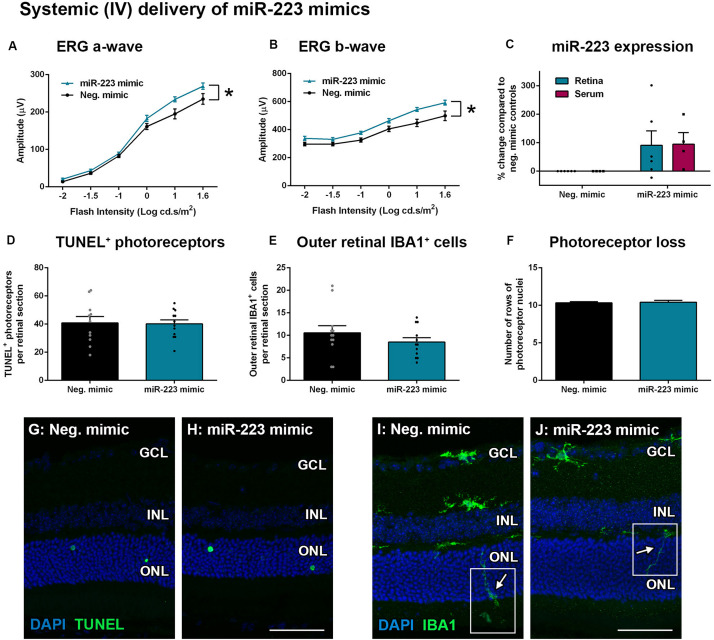
Systemic intravenous (IV) delivery of miR-223 mimics during 5 days of photo-oxidative damage. **(A,B)** WT animals injected with miR-223 mimics again had a significantly higher a-wave **(A)** and b-wave **(B)** than negative controls (*P* < 0.05), as assessed by electroretinography (ERG). **(C)** Retinal and serum expression of miR-223 were not significantly changed after miR-223 mimic injections (*P* > 0.05). **(D,E)** There was no significant difference in TUNEL^+^ **(D)** or IBA1^+^ cells in the outer retina **(E)** between groups (*P* > 0.05). **(F)** No change in photoreceptor cell rows was observed between groups (*P* > 0.05). **(G,H)** Representative images depicting TUNEL^+^ photoreceptors (green). **(I,J)** Representative images depicting IBA1 immunohistochemistry (green), with arrows pointing toward the processes of IBA1^+^ cells in the outer retina (white boxes). GCL, ganglion cell layer; INL, inner nuclear layer; ONL, outer nuclear layer. Scale bar is 50 μm. Sample size is *N* = 4–6. *Denotes significance (*P* < 0.05).

Wild-type animals were also injected systemically (IV) with miR-223 mimics (0.5 mg/kg) at 2 days into a 5-day photo-oxidative damage protocol. This time point was chosen for the IV injections as Invivofectamine 3.0 is thought to have a shorter half-life in circulation compared to the eye, where retinal transfection has been shown to last 3–4 days (Chu-Tan et al., 2020). At 5 days photo-oxidative damage, the ERG a-wave and b-wave were significantly increased after IV injection with the miR-223 mimic, compared to negative controls (Figures 6A,B, *P* < 0.05). When assaying both the retina and the serum for miR-223 levels, there was no significant difference between miR-223 mimic-injected animals and negative controls (Figure 6C, *P* > 0.05). There was also no difference in TUNEL^+^ photoreceptors (Figures 6D,G,H, *P* > 0.05), outer retinal IBA1^+^ microglia and macrophages (Figures 6E,I,J, *P* > 0.05), or number of photoreceptor cell rows (Figure 6F, *P* > 0.05), indicating no major differences in histology following injections.

These data demonstrate that delivery of miR-223 mimics to the retina via local (IVT) or systemic (IV) injection may improve the retinal response. However, only local delivery of miR-223 may be effective in providing protection to the photoreceptor cell layer.

## Discussion

In this study, we identified several key roles of miR-223 in the healthy and degenerating retina. First, we found that miR-223 is required for the maintenance of healthy retina, with deficiency in miR-223 causing aberrant retinal function. Secondly, we found that although retinal, circulating, and s-mEV miR-223 are all upregulated in response to retinal degeneration, miR-223 expression is reduced in microglia and macrophages. A deficiency in miR-223 in both retinal and circulating immune cells in degeneration may alter the expression of key inflammatory genes, including *Ctse*. Finally, we demonstrated that local delivery of a miR-223 mimic may reduce photoreceptor loss and improve retinal function. Overall, these data implicate miR-223 in the retina as having important roles in influencing both retinal function and inflammation.

### miR-223 Is Required for Maintaining Retinal Function

In dim-reared miR-223^–/–^ animals, our data demonstrated that absence of miR-223 had an adverse effect on the retinal function of both photoreceptors (a-wave) and the ON-bipolar and Müller cells (b-wave), as well as minor photoreceptor loss. miR-223 deficiency led to a small reduction in retinal *C1qa*, a classical complement activator produced by microglia that is thought to be critically involved in synapse elimination by phagocytosis (Stevens et al., 2007) and maintaining inner nuclear layer (INL) integrity (Mukai et al., 2018). It is possible that reduced *C1qa* in miR-223-null retinas contributes to altered retinal function in normal conditions; although as *C1qa* is not a known target of miR-223, this could be occurring through other microglial components that may activate the complement system. Other studies have suggested a role for miR-223 in the neuroprotection of photoreceptors and second-order neurons, showing that the expression of glutamate receptor (GluR) subunits may be regulated by miR-223 (Harraz et al., 2012; Morquette et al., 2019). In particular, GluR2 and NR2B have been identified as potential targets of miR-223 (Harraz et al., 2012), with GluR2 thought to be expressed by bipolar cells housed within the INL (Lin et al., 2012). miR-223 *in situ* hybridization in an EAU model has indicated that in addition to being expressed within the perivascular lesion and in subretinal inflammatory cells, miR-223 may be also expressed within the INL (Watanabe et al., 2016). In the miR-223^–/–^ retina, it is possible that the absence of miR-223 may lead to dysregulation of GluR density on bipolar cells, decreasing their threshold of depolarization and inducing excitotoxicity. This may cause a reduced b-wave and increased gliosis, both of which were observed in the miR-223^–/–^ mice in degenerating conditions. Further studies could investigate the interactions between miR-223 and these GluR subunits, as well as any potential expression of miR-223 by bipolar cells in the retina. This may contribute toward an understanding of whether miR-223-mediated neuroprotection plays a role in modulating retinal function and neuronal survival.

### Cathepsin E Is Upregulated During miR-223 Deficiency

We identified *Ctse* [a predicted target of miR-223 (Gantier, 2013)] as a key inflammatory gene that was heavily upregulated in all miR-223-null retinas and isolated CD11b^+^ cells in this study. CTSE is an aspartic proteinase known to mediate a range of critical macrophage functions including lysosomal activity, autophagy, and recruitment (Yamamoto et al., 2012). As miR-223 deficiency would likely affect *Ctse* as it is a predicted target, its upregulation in the retina could contribute to macrophage dysregulation. Alternatively, CTSE has been previously thought to contribute to neuronal degeneration during aging and may be expressed by aging neurons during degeneration (Nakanishi et al., 1994; Nishishita et al., 1996). In human retinas, increasing neuronal expression of CTSE was evident with age, in addition to its expression in immune cells and some Müller cells (Bernstein et al., 1998). Interestingly, excessive stimulation of GluR in hippocampal neurons has been shown to induce *Ctse* gene expression in these neurons, in addition to activated microglia (Tominaga et al., 1998). It is possible that interactions between miR-223, GluR, and *Ctse* could be taking place in retinal neurons (including bipolar cells) as well, as we have indicated that miR-223 can be expressed in neuronal cell types (photoreceptors). As hypothesized above, the altered b-wave response associated with miR-223^–/–^ mice may be due to GluR signaling and excitotoxicity. If high neuronal and microglial *Ctse* expression is stimulated by GluR dysregulation in miR-223^–/–^ mice, it is possible that *Ctse* may be contributing to neuronal degeneration under normal conditions. Accordingly, we observed photoreceptor loss and a reduced a-wave in dim-reared miR-223^–/–^ mice. However, in photo-oxidative damage, we did not observe photoreceptor degeneration in the miR-223^–/–^ mice, perhaps indicating that other inflammatory functions of *Ctse* were more imperative in this scenario.

Although beyond the scope of this preliminary investigation into the relationship between miR-223 and *Ctse*, further studies are needed to investigate whether retinal levels of CTSE protein are altered in miR-223^–/–^ or miR-223-supplemented mice and to determine what roles CTSE may have in influencing neuronal function or inflammation in the retina.

### miR-223 Regulates the Inflammatory Profile of Microglia and Macrophages

We identify retinal microglia and macrophages as an abundant source of miR-223, and their levels decrease substantially in degenerating conditions. As miR-223 is a critical inhibitor of many inflammatory processes, including the NLRP3 inflammasome (Bauernfeind et al., 2012; Yang et al., 2015; Neudecker et al., 2017b), cathepsin activity (Gantier, 2013), chemokine signaling (Dorhoi et al., 2013), the NF-κB pathway (Zhou et al., 2018), and JAK/STAT signaling (Chen et al., 2012), a reduction in miR-223 would likely cause higher inflammation within these cells. As discussed earlier, we observed that *Ctse* was substantially increased in CD11b^+^ cells from both the retina and the blood of miR-223-null mice. We also found that in miR-223-null CD11b^+^ cells from the retina, there was increased *C1qa* which is associated with progressive retinal degeneration (Silverman et al., 2016; Jiao et al., 2018), as well as increased *Stat3*, also associated with retinal disease (Chen et al., 2016, 2019). In the blood of miR-223^–/–^ mice, circulating CD11b^+^ cells showed an increase in *C3* and *Il-1*β, which are both critically linked to macrophage-mediated retinal degeneration (Rutar et al., 2011a; Hu et al., 2015; Eandi et al., 2016; Natoli et al., 2017a, b).

Additionally, we observed that there appeared to be skewing in the circulating leukocyte population of miR-223-null animals toward a higher proportion of CD11b^+^ cells, indicating that the cells may be producing more pro-inflammatory factors. There is also a possibility that CD11b^–^ cells may have been lost with miR-223 deficiency. We note that a study by Johnnidis et al. (2008) indicated that miR-223-null mice have an expanded granulocytic compartment, leading to neutrophil hyperactivity. However, Galloway et al. (2019) found that although miR-223-null mice showed a similar pro-inflammatory phenotype to WT mice, an absence of miR-223 hindered anti-inflammatory functions and effective M2 (CD206^+^) polarization. Although some differences have been observed between these studies, the commonality is that miR-223 is required for normal myeloid cell activation and differentiation and that an absence of miR-223 alters the inflammatory profile to a state that may exacerbate inflammatory conditions, including retinal degenerative diseases. Further investigation into which circulating cells of the hematopoietic lineage may be affected by miR-223 deficiency may uncover therapeutic targets for modulation.

### Dual Roles for miR-223 in Retinal Degeneration

Although retinal and circulating CD11b^+^ cells from photo-oxidative damage were more pro-inflammatory in response to miR-223 deficiency, this did not translate to a more damaging outcome for the retina in knockout mice. We demonstrated that miR-223-null mice had retained more photoreceptors, accompanied by fewer outer retinal microglia and macrophages. In addition, with the exception of *Ctse*, there were no changes in any inflammatory genes assayed in the retinas of miR-223-null mice, several of which are targets of miR-223. It is possible that some of the protective functions of *Ctse* might be at play here, such as maintaining autophagic proteolysis (Tsukuba et al., 2013). This could occur in the context of photoreceptor outer segment phagocytosis, a known function of a related molecule, cathepsin D (Rakoczy et al., 2002). It is possible that miR-223 deficiency has a role in the protection of the retina during damage through the promotion of anti-inflammatory mechanisms or protective responses. Li et al. (2019) proposed that miR-223-deficient microglia appeared to remain in a resting state and retained their autophagic functions in experimental autoimmune encephalomyelitis (EAE), reducing CNS inflammation and pathology. It is possible that autophagy, which may be influenced by photo-oxidative damage (Chen et al., 2013), is hindered with miR-223 signaling in degenerating conditions. Further studies are required to address autophagy and cathepsins as a potential mechanism involved in miR-223 interactions in the retina, including autophagic photoreceptor cell death. The concept of miR-223 having alternative damaging roles has also been explored in a rabbit glaucoma model, where miR-223 mimics induced apoptosis and inflammation in retinal ganglion cells by targeting HSP-70 (Ou-Yang et al., 2020). In another study, miR-223 targeted FOXO3, positively regulating pathogenic Th17 cells in an EAU model (Wei et al., 2019).

However, miR-223-null photo-oxidative damaged mice exhibited increased GFAP, indicating a higher level of reactive gliosis and retinal cell stress. The ERG b-wave was also dampened in miR-223-null photo-oxidative damaged mice, indicating reduced ON-bipolar and Müller cell activity. A potential loss of miR-223-mediated neuroprotection could again have contributed to both of these observations (Harraz et al., 2012; Morquette et al., 2019). These data point to miR-223 having dual protective and damaging roles in the degenerating retina.

### Delivery of miR-223 Mimics Improve Retinal Function

Without miR-223, retinal function appears to be worsened, whereas both locally and systemically delivered miR-223 mimics led to an improvement in retinal function. This again demonstrates that miR-223 may be required in the preservation of the retinal response, perhaps by promoting microglial homeostatic maintenance (Galloway et al., 2019; Guo et al., 2019), or through neuroprotection and GluR signaling as discussed earlier (Harraz et al., 2012; Morquette et al., 2019). When delivered locally, miR-223 supplementation also protected mice from photoreceptor loss. The discrepancy between miR-223^–/–^ and miR-223-supplemented mice regarding photoreceptor layer morphology in degeneration could be a limitation of using miR-223-null mice with the photo-oxidative damage model. It is possible that any photoreceptor degeneration in miR-223-null mice in normal conditions may lead to a pre-conditioning effect against oxidative stress and inflammation caused by retinal damage.

However, given that IVT delivery increased retinal miR-223 by ∼10,000%, it was somewhat surprising that the IV injection also led to an improved retinal response, even though no detectable increase in retinal miR-223 was observed at the time of tissue collection. It is possible that the amount of miR-223 delivered to animals through IV delivery was sufficient to see an effect on retinal function; however, more sustained delivery to increase the bioavailability in the blood might be required to attain a measurable effect in the photoreceptor layer. It is also possible that the circulating immune cells had taken up the systemic-injected miR-223 mimics and were altered prior to retinal infiltration, shifting them toward an anti-inflammatory state (Galloway et al., 2019) and potentially reducing pathogenic mechanisms that may adversely affect retinal function. As miR-223 mimics have been useful in reducing pathogenesis in CNS studies (Shi et al., 2013; Yang et al., 2015; Ding et al., 2018; Galloway et al., 2019), identifying how miR-223 mimics modulate the retinal response or inflammation (including *Ctse*) will be key to determining any therapeutic potential of manipulating miR-223 levels in the retina and circulation. In further studies investigating alternative IV doses and injection paradigms of miR-223 mimics, system-wide organ pathology and circulating immune profiles should be monitored following systemic treatments. In addition, fluorescent tracing studies of miR-223 mimics in circulation may assist in determining both retinal and systemic cell transfection.

### miR-223 as a Circulating miRNA in Retinal Degeneration

We determined that retinal miR-223 levels were upregulated during degeneration in the photo-oxidative damage model, corroborating previous findings using a range of retinal disease models (Fuller-Carter et al., 2015; Saxena et al., 2015; Chung et al., 2016; Watanabe et al., 2016; Huang et al., 2017). This may have occurred either through increased photoreceptor expression of miR-223, or the accumulation of large numbers of miR-223-expressing recruited macrophages in areas of retinal degeneration (Natoli et al., 2017a). We also identified circulating serum levels of miR-223 to also be upregulated in photo-oxidative damage. miR-223 has previously been identified as a circulating biomarker in a range of chronic inflammatory conditions (Zhou et al., 2015; Wang et al., 2016; Cisilotto et al., 2020), including neurological disorders (Fenoglio et al., 2013; Wang et al., 2014). In retinal degeneration, it is possible that elevated miR-223 levels in the circulation may be an indication of disease, as occurs in neovascular AMD patient plasma (Ertekin et al., 2014) and in peripheral blood cells (Litwinska et al., 2019). An increase in systemic miR-223 could have occurred due to elevation in the retina in response to light exposure, which has been previously shown to alter retinal miRNA expression levels (Krol et al., 2010). Excess miR-223 may have leeched out of the retina into the blood. Alternatively, miR-223 may have been upregulated in circulating immune cells in response to light exposure.

Interestingly, miR-223 may also participate in exosomal communication in neurological disorders, including stroke and dementia (Chen et al., 2017; Wei et al., 2018). Shurtleff et al. (2016) demonstrated that miR-223 was a specific highly abundant exosome cargo in HEK293T cells, where Y-box protein 1 (YBX1) was required for selective sorting of specific miRNAs (including miR-223) into exosomes. YBX1 has recently been proposed as a central transcription factor involved in aging (Ma et al., 2020). In retinal degeneration, it is possible that miR-223-loaded s-mEVs (including exosomes) from the retina could leech out into the circulation, or s-mEVs could be produced systemically. In the current study, we found that s-mEVs isolated from the retina during degeneration had significantly increased miR-223 content. However, the question remains as to which cells produce these miR-223-loaded s-mEVs in the retina, and whether leeching into the circulation would have any systemic effects. In other diseases, macrophage-derived extracellular vesicles carrying miR-223 have been shown to supplement epithelial ovarian cancer cells with miR-223 (Zhu et al., 2019), while neutrophil-derived extracellular vesicles act to increase miR-223 in pulmonary epithelial cells (Neudecker et al., 2017a). Hence, further investigation into which cells produce these miR-223-loaded s-mEVs in the retina will help to elucidate mechanisms by which s-mEV-derived miR-223 exerts local and systemic responses that influence the progression of retinal degeneration.

## Conclusion

We demonstrate in this study that miR-223 is required for maintenance of the healthy retina, through its regulation of retinal function and modulation of inflammatory molecules by retinal and circulating immune cells. We show that miR-223 mimics may be effective in improving the retinal response to photo-oxidative damage and demonstrate that local miR-223 delivery can achieve a beneficial effect in the preservation of the photoreceptor layer during retinal degeneration. As miR-223 may target hundreds of genes and several biological pathways, further understanding the interactions of miR-223 in the retina will be critical in defining the therapeutic utility of miR-223 in retinal degenerative diseases.

## Data Availability Statement

All datasets presented in this study are included in the article/supplementary material.

## Ethics Statement

The animal study was reviewed and approved by the Australian National University (ANU) Animal Experimentation Ethics Committee (Ethics ID: A2017/41).

## Author Contributions

NF designed the study, conducted experiments and analysis, and wrote the manuscript. JW, SD, CDi, RA-B, AC, YW, and CN conducted experiments and/or analysis. JW, SD, RA-B, AC, YW, JC-T, US, RE, and SR revised the manuscript. CDo and SR managed the miR-223^–/–^ animals and contributed to the study design. RE, SM, and JP designed the study. RN supervised and designed the study, conducted experiments and analysis, and revised the manuscript. All authors contributed to the article and approved the submitted version.

## Conflict of Interest

The authors declare that the research was conducted in the absence of any commercial or financial relationships that could be construed as a potential conflict of interest.
